# Safety and feasibility of ultrasound-guided placement of peripherally inserted central catheter performed by neurointensivist in neurosurgery intensive care unit

**DOI:** 10.1371/journal.pone.0217641

**Published:** 2019-05-31

**Authors:** Yong Oh Kim, Chi Ryang Chung, Eunmi Gil, Chi-Min Park, Gee Young Suh, Jeong-Am Ryu

**Affiliations:** 1 Department of Critical Care Medicine, Samsung Medical Center, Sungkyunkwan University School of Medicine, Seoul, Republic of Korea; 2 Department of Surgery, Samsung Medical Center, Sungkyunkwan University School of Medicine, Seoul, Republic of Korea; 3 Division of Pulmonary and Critical Care Medicine, Department of Medicine, Samsung Medical Center, Sungkyunkwan University School of Medicine, Seoul, Republic of Korea; 4 Department of Neurosurgery, Samsung Medical Center, Sungkyunkwan University School of Medicine, Seoul, Republic of Korea; Cleveland Clinic, UNITED STATES

## Abstract

We evaluated the safety and feasibility of ultrasound-guided peripherally-inserted central venous catheters (PICC) by a neurointensivist at the bedside compared to fluoroscopy-guided PICC and conventional central venous catheter (CCVC). This was a retrospective study of adult patients who underwent central line placement and were admitted to the neurosurgical intensive care unit (ICU) between January 2014 and March 2018. In this study, the primary endpoint was central line-induced complications. The secondary endpoint was initial success of central line placement. Placements of ultrasound-guided PICC and CCVC performed at the bedside if intra-hospital transport was inappropriate. Other patients underwent PICC placement at the interventional radiology suite under fluoroscopic guidance. A total of 191 patients underwent central line placement in the neurosurgery ICU during the study period. Requirement for central line infusion (56.0%) and difficult venous access (28.8%) were the most common reasons for central line placement. The basilic vein (39.3%) and the subclavian vein (35.1%) were the most common target veins among patients who underwent central line placement. The placements of ultrasound-guided PICC and CCVC at the bedside were more frequently performed in patients on mechanical ventilation (*p* = 0.001) and with hemodynamic instability (*p* <0.001) compared to the fluoroscopy-guided PICC placement. The initial success rate of central line placement was better in the fluoroscopy-guided PICC placement than in the placements of ultrasound-guided PICC and CCVC at the bedside (*p* = 0.004). However, all re-inserted central lines were successful. There was no significant difference in procedure time between the three groups. However, incidence of insertional injuries was higher in CCVC group compared to PICC groups (*p* = 0.038). Ultrasound-guided PICC placement by a neurointensivist may be safe and feasible compared to fluoroscopy-guided PICC placement by interventional radiologists and CCVC placement for neurocritically ill patients.

## Introduction

Neurocritically ill patients and neurosurgery patients often require access to the central vein during their intensive care unit (ICU) stay [1]. Peripherally-inserted central venous catheters (PICC) have been widely used for central venous access for long-term intravenous therapy [2]. PICCs have been increasingly utilized because of easy placement and a lower rate of insertion-related mechanical complications, venous thrombosis, and infection [1–5]. Therefore, the use of PICC has many theoretical advantages in the neuroscience ICU setting due to the low risk of complications [3,4].

Traditionally, PICC placement has been performed by interventional radiologists in the interventional radiology suite under fluoroscopic guidance [2,6,7]. However, critically ill patients can experience adverse events during transport if they have respiratory failure or hemodynamic instability [8–10]. Recently, ultrasound-guided PICC has been performed by vascular access teams [1,4,11–13]. It has been also performed by intensivists at the bedside for critically ill patients who were transport risks [8]. However, there is limited data on the safety and feasibility of ultrasound-guided PICC performed by neurointensivists in the neurosurgery ICU compared to fluoroscopy-guided PICC or conventional central venous catheter (CCVC). Therefore, the objective of this study was to investigate the safety and feasibility of ultrasound-guided PICC by a neurointensivist at the bedside compared to fluoroscopy-guided PICC and CCVC.

## Materials and methods

### Study population

**Fig 1:**
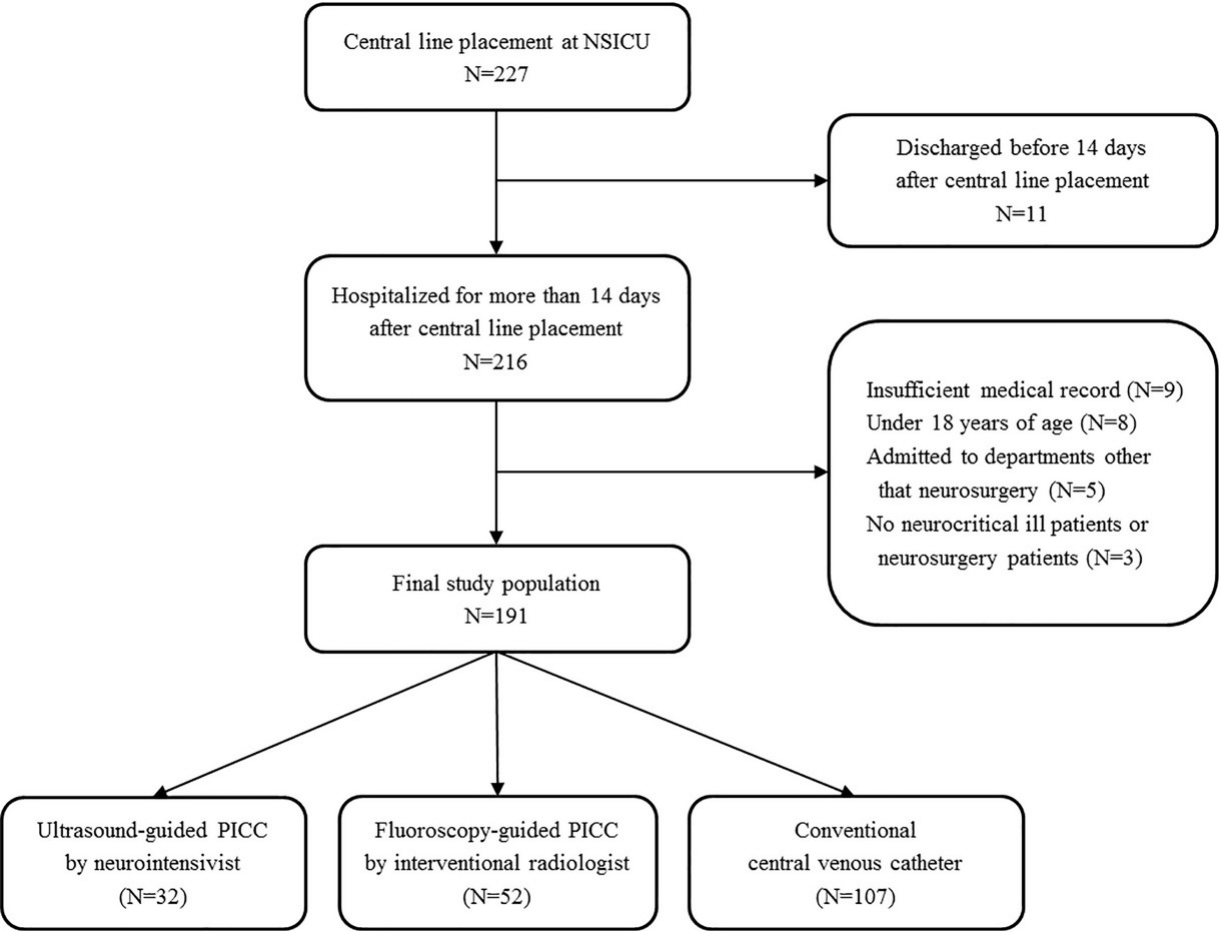
Study flow chart. NSICU, neurosurgery intensive care unit; PICC, peripherally-inserted central venous catheter.

This was a retrospective and observational study of adult patients admitted to the neurosurgical ICU at Samsung Medical Center between January 2014 and March 2018. This study was approved by the Institutional Review Board of Samsung Medical Center (SMC 2018-09-011). The requirement for informed consent was waived due to its retrospective nature. Clinical and laboratory data were collected by a trained study coordinator using a standardized case report form. Adult patients admitted to the neurosurgical ICU who underwent placement of PICC or CCVC during the study period were included in the study. The patient list was cross-referenced with the electronic order entry system and the electronic medical record to identify all patients who underwent placement of PICC or CCVC during their ICU stay. Those hospitalized for more than 14 days after central line insertion were selected. Of these patients, patients under age 18, those discharged before 14 days after central line insertion, and those with insufficient medical records were excluded. In addition, patients were excluded if they were admitted to departments other that neurosurgery or had no neurological problems. A total of 191 patients were analyzed in this study (Fig 1).

### Definitions and outcomes

We retrospectively reviewed all placement of PICC and CCVC in neurocritically ill patients and neurosurgery patients admitted to the neurosurgery ICU during the study period. All patients underwent preliminary chart review to ensure a central line had been placed and the following baseline demographics were abstracted from the electronic medical chart: patient’s age and comorbidities, causes of ICU admission, severity scores on ICU admission, indication for central line placement, laboratory data on central line placement, duration of the dwell time for the device, indication for ultrasound of the upper extremity, and causes of removal. As associated data for the insertion procedure, we investigated initial success of central line placement, malposition, re-insertion, catheter tip position, and procedure time. In addition, we investigated complications at insertion or during maintenance, such as insertional injury, bleeding or hematoma, suspected infected catheters, central line-associated bloodstream infections (CRBSI), and symptomatic central line-related venous thrombosis. CLABSI was defined as identification of the same bacteria cultured from the line and from one or more blood cultures not drawn from the line, with no other identifiable source for the bloodstream infection [1,14,15]. For patients with positive blood cultures, a chart review was done to identify the source of the bacteremia or candidemia. CLABSI were also identified by cross-reference with CLABSI monitoring of the Infection Prevention and Control Team of Samsung Medical Center. Early and late-onset infections were defined as occurring seven days or less, or greater than seven days after central line placement [16]. Symptomatic PICC-related large vein thrombosis was determined by reviewing the duplex ultrasound reports for every patient who had a PICC placed [1]. Ultrasound examination (compression ultrasonography and duplex Doppler) was performed only when clinically indicated by signs and symptoms suggesting venous occlusion (edema of the arm, PICC malfunction, unexplained local pain, etc.) [4]. This was defined as proximal large vein thrombosis of the ipsilateral extremity, confirmed to be associated with the PICC or confirmed within five days of PICC removal [17]. ICU-free days were defined as the number of days between successful transfer to a normal ward and 28 days after study enrollment. Therefore, the number of ICU-free days was 0 if the patient died before day 28 or stayed in the ICU for 28 days or longer [18,19].

In this study, the primary endpoint was central line-induced complications. The secondary endpoint was initial success of central line placement.

### Procedure

A neurointensivist or neurosurgeon determined central line placement. The ultrasound-guided PICC or CCVC was preferred at the bedside for the patients with hemodynamic instability, on mechanical ventilation or those with neurocritical conditions. PICCs were performed by an interventional radiologist in the interventional radiology suite under fluoroscopic guidance if bedside insertion was inappropriate. In this study, indications for placement of PICC and CCVC were the need for a central line for parenteral nutrition, infusion of drugs requiring a central line (pH <5 or >9, osmolarity >500 mOsm/l, drugs associated with endothelial damage), need for frequent blood sampling, or difficult venous access. Although all PICCs were inserted as an elective procedure, CCVC placement was mainly inserted as an emergency procedure. Contraindications to PICC placement were small deep veins of the arm (diameter of target vein <3 mm), local contraindications due to specific arm conditions, such as skin infection or burns, as well as actual or impending chronic renal failure requiring an arteriovenous fistula. Severe arm edema or obesity was not considered contraindications for PICC placement. There was no specific contraindication of CCVC placement. We used 5 Fr single-lumen silastic catheters or 5 Fr dual-lumen Turbo-Ject Power-Injectable PICCs (Cook, Bloomington, MN, USA), 5 Fr triple-lumen PowerPICC Catheters (Bard Access Systems, Salt Lake City, UT, USA), or 6 Fr dual-lumen Vaxel PICCs with PASV Valves (Navilyst, Marlborough, MA, USA). We also used 7 Fr dual-lumen Blue FlexTip ARROWgard Blue, 18Ga, (Arrow International, Inc. USA), 7 Fr triple-lumen Blue FlexTip ARROWgard Blue, 18Ga, (Arrow International, Inc. USA), 11.5 Fr dual-lumen Mahurkar catheter (Quinton Instrument, Bothell, Wash., USA) sterile dual-lumen catheter kit, 13 Fr. Triple-lumen Power‐Trialysis catheter (Bard Access Systems, Salt Lake City, UT, USA). Peripheral venous access was obtained through the basilic, the brachial, or the cephalic vein. Central venous access was obtained through the subclavian, the internal jugular, or the femoral vein. The PICC catheterization inserted by a neurointensivist was performed by ultrasound-guided puncture of the deep veins in the upper mid-arm using the micro-introducer technique. A standard 5 to 10 MHz linear ultrasound probe was used. Maximal barrier precautions were consistently used during the procedure (cap, mask, sterile gown, sterile gloves, vast sterile field). The length of the PICC was determined as the distance from the puncture site through the humeral head to the sternal notch and to the third intercostal space. The correct position of the tip of the catheter (that is, in proximity of the junction between the superior vena cava and the right atrium) was verified by chest radiographs. If it was to be used immediately or re-inserted due to malposition, an agitated saline (a solvent that quickly and repeatedly moves the saline between the two syringes to dissolve micro air bubbles in the saline) was used to confirm flow of blood into the right atrium by ultrasonography. In addition, the correct position of the tip of the catheter was verified during the procedure using intracavitary electrocardiography if the agitated saline method was inappropriate [4]. Malposition was defined if the catheter tip is not located at the cavoatrial junction in chest radiographs. If the catheterization was performed by an interventional radiologist, nonionic contrast medium (30 mL) was injected through the intravenous line, and the opacified vein was punctured with a 21-gauge micropuncture needle under fluoroscopic guidance [20]. The cephalic vein was used only in cases where the basilic vein was invisible or non-opacified despite squeezing or shifting the contrast medium flow to the medial side arm [20]. The position of the catheter tip was immediately checked using fluoroscopy. The CVC catheterization inserted by a neurointensivist or neurosurgical resident. Although catheterization via subclavian vein was performed blindly using anatomic landmarks as a guide to vessel position, catheterization via internal jugular vein or femoral vein was performed by ultrasound-guided puncture. Maximal barrier precautions were also used consistently during the CCVC procedure. Dressing changes occurred every seven days, or if soiled in all placements.

### Statistical analyses

All data are presented as medians and interquartile ranges (IQRs) for continuous variables and numbers (percentages) for categorical variables. Data were compared using the Kruskal-Wallis test and the Mann-Whitney U test for continuous variables and the Chi-squared test or Fisher’s exact test for categorical variables. All tests were two-sided and *p* values < 0.05 were considered statistically significant. Data were analyzed using IBM SPSS statistics version 20 (IBM, Armonk, NY, USA).

## Results

### Baseline characteristics and procedural characteristics

A total of 191 patients underwent central line placement in the neurosurgery ICU during the study period. The median age of the patients was 59.0 (IQR: 44.5–66.5) years. Of 191 patients, 82 (42.9%) were males. Malignancies (73.8%) and hypertension (48.7%) were the most common comorbidities among patients who underwent central line placement. Elective surgery for brain tumors (51.3%) and intracranial hemorrhages (17.3%) were the most common reasons for ICU admission. The APACHE II score on ICU admission was higher in the CCVC group compared to the other groups. There were no significant differences in age, gender, body mass index, comorbidities, or GCS on ICU admission between the three groups (Table 1).

**Table 1:** Baseline characteristics.

		Ultrasound-guided PICC by neurointensivist (n = 32)	Fluoroscopy-guided PICC by interventional radiologist (n = 52)	Conventional central venous catheter (n = 107)	*p* value
Age (yr)—median (IQR)		64.5 (51.5–71.0)	59.0 (45.5–66.5)	57.0 (43.0–64.5)	0.093
Gender, male—no. of patients (%)		12 (37.5)	17 (32.7)	53 (49.5%)	0.105
BMI (kg/m^2^)—median (IQR)		23.7 (21.6–25.8)	23.7 (20.2–27.3)	23.8 (21.6–25.9)	0.809
Obese (BMI >30kg/m^2^)—no. of patients (%)		1 (3.1)	5 (9.6)	9 (8.4%)	0.533
Comorbidities—no. of patients (%)					
	Malignancy	23 (71.9)	39 (75.0)	79 (73.8)	0.951
	Hypertension	18 (56.2)	26 (50.0)	49 (45.8)	0.569
	Diabetes mellitus	11 (34.4)	14 (26.9)	30 (28.0)	0.739
	Previous stroke	23 (71.9)	24 (46.2)	53 (49.5)	0.049
	Dyslipidemia	8 (25.0)	8 (15.4)	20 (18.7)	0.548
Cause of ICU admission—No. of patients (%)					0.027
	Brain tumor	17 (53.1)	34 (65.4)	47 (43.9)	
	Intracranial hemorrhage	6 (18.8)	8 (15.4)	19 (17.8)	
	Traumatic brain injury	2 (6.2)	2 (3.8)	16 (15.0)	
	Subarachnoid hemorrhage	4 (12.5)	0 (0)	9 (8.4)	
	Cerebral infarction	1 (3.1)	1 (1.9)	4 (3.7)	
	CNS infection	0 (0)	3 (5.8)	0 (0)	
	Other	2 (6.2)	4 (7.7)	12 (11.2)	
GCS on ICU admission—median (IQR)		5.0 (3.0–10.0)	6.0 (3.0–9.0)	3.0 (3.0–8.0)	0.191
APACHE II score on ICU admission—median (IQR)		19.0 (16.0–24.0)	19.0 (14.5–22.0)	22.0 (17.0–26.0)	0.009

PICC, peripherally-inserted central venous catheter; IQR, interquartile range; BMI, body mass index; ICU, intensive care unit; CNS, central nerve system; GCS, Glasgow Coma Scale; APACHE, Acute Physiology and Chronic Health Evaluation

Requirement for central line infusion (56.0%) and difficult venous access (28.8%) were the most common reasons for central line placement. PICC placement was performed after removal of CCVC in 19 (9.9%) patients. The basilic vein (39.3%) and the subclavian vein (35.1%) were the most common target veins among patients who underwent central line placement. The placements of ultrasound-guided PICC and CCVC at the bedside were more frequently performed in patients on mechanical ventilation (*p* = 0.001) and with hypotension (*p* <0.001) compared to fluoroscopy-guided PICC placement (Table 2).

**Table 2:** Procedural characteristics of central line placement.

		Ultrasound-guided PICC by neurointensivist (n = 32)	Fluoroscopy-guided PICC by interventional radiologist (n = 52)	Conventional central venous catheter (n = 107)	*p* value
Reason for central line placement—No. of patients (%)					<0.001
	For infusion of drugs requiring a central line	11 (34.4)	11 (21.2)	85 (79.4)	
	Difficult venous access	17 (53.1)	36 (69.2)	2 (1.9)	
	Dialysis	0 (0)	0 (0)	19 (17.8)	
	Parenteral nutrition	2 (6.2)	3 (5.8)	1 (0.9)	
	Frequent blood sampling	2 (6.2)	2 (3.8)	0 (0)	
Anticoagulation—No. of patients (%)		0 (0)	5 (9.6)	2 (1.9)	0.025
Use of antiplatelet agents—No. of patients (%)		0 (0)	0 (0)	4 (3.7)	0.201
Use of mechanical ventilator—No. of patients (%)		25 (78.1)	16 (30.8)	67 (62.6)	0.001
Use of renal replacement therapy—No. of patients (%)		0 (0)	1 (1.9)	19 (17.8)	0.001
Invasive ICP monitoring—No. of patients (%)		10 (31.2)	14 (26.9)	50 (46.7)	0.859
Mean BP on central line placement (mmHg)—median (IQR)		62.7 (55.7–69.5)	74.3 (69.3–79.0)	66.3 (54.5–73.3)	<0.001
Hypotension (systolic BP <90 mmHg)—No. of patients (%)		15 (46.9)	5 (9.6)	51 (47.7%)	<0.001
Insertion direction (right)—No. of patients (%)		18 (56.2)	19 (36.5)	85 (79.4%)	<0.001
Placement in paretic arm—No. of patients (%)		5 (15.6)	11 (21.2)		0.531
Diameter of catheter—No. of patients (%)					0.039
	5 Fr	27 (84.4)	33 (63.5)	0 (0)	<0.001
	6 Fr	5 (15.6)	19 (36.5)	0 (0)	
	7 Fr	0 (0)	0 (0)	88 (82.2%)	
	11.5 Fr	0 (0)	0 (0)	13 (12.1)	
	13 Fr	0 (0)	0 (0)	6 (5.6)	
Insertion site—No. of patients (%)					<0.001
	Basilic vein	26 (81.2)	49 (94.2)	0 (0)	
	Brachial vein	4 (12.5)	0 (0)	0 (0)	
	Cephalic vein	2 (6.2)	3 (5.8)	0 (0)	
	Subclavian vein	0 (0)	0 (0)	67 (62.6)	
	Internal jugular vein	0 (0)	0 (0)	18 (16.8)	
	Femoral vein	0 (0)	0 (0)	22 (20.6)	
Number of catheter lumen—No. of patients (%)					<0.001
	1	0 (0)	32 (61.5)	0	
	2	29 (90.6)	19 (36.5)	14 (13.1)	
	3	3 (9.4)	1 (1.9)	93 (86.9)	
Coagulation results on the day of central line placement—median (IQR)					
	Platelet count (×10^3^/μl)	198.0 (141.5–322.0)	237.0 (171.5–369.0)	151.0 (104.5–213.0)	<0.001
	PT(INR)	1.1 (1.1–1.2)	1.1 (1.1–1.2)	1.2 (1.1–1.4)	<0.001
	aPTT (sec)	37.9 (34.3–44.1)	37.5 (32.6 (43.9)	38.0 (33.9–45.2)	0.870

PICC, peripherally inserted central catheter; ICP, intracranial pressure; BP, blood pressure; IQR, interquartile range; INR, international normalized ratio; aPTT, activated partial thromboplastin time

#### Clinical outcomes

The initial success rate of central line placement was better in the fluoroscopy-guided PICC placement than in the placements of ultrasound-guided PICC and CCVC at the bedside (*p* = 0.004). Indeed, malposition and re-insertion were more common in the placements of ultrasound-guided PICC and CCVC than the fluoroscopy-guided PICC placement (*p* = 0.005). However, all re-inserted central lines were successful. There were no significant differences in procedure times between the three groups (Table 3).

**Table 3:** Clinical outcomes.

			Ultrasound-guided PICC by neurointensivist (n = 32)	Fluoroscopy-guided PICC by interventional radiologist (n = 52)	Conventional central venous catheter (n = 107)	*p* value
Procedural data						
	Initial success of central line insertion—No. of patients (%)		26 (81.2)	52 (100)	87 (81.3)	0.004
	Malposition & re-insertion—No. of patients (%)		6 (18.8)	0 (0)	20 (18.7)	0.005
		Success of re-insertion	6 (18.8)		20 (18.7)	
		Use as midline catheter	3 (9.4)			
	Procedure time (min)—median (IQR)		20.0 (15.0–40.0)	15.0 (14.0–23.5)	19.0 (14.0–35.0)	0.236
Duration of using central line (day)—median (IQR)			20.3 (9.7–35.3)	28.0 (16.2–34.0)	9.0 (5.0–14.0)	<0.001
Complications—No. of patients (%)						
	CLABSI		2 (6.2)	0 (0)	1 (0.9)	0.060
	Symptomatic central line-related venous thrombosis		2 (6.2)	0 (0)	0 (0)	0.068
	Insertional injury		0 (0)	0 (0)	8 (7.5)	0.038
		Lung injury	0 (0)	0 (0)	4 (3.7)	
		Moderate or severe bleeding and hematoma	0 (0)	0 (0)	4 (3.7)	
Cause of removal—No. of patients (%)						<0.001
	Unnecessary		14 (43.8)	35 (67.3)	69 (64.5)	
	Fever		8 (25.0)	7 (13.5)	15 (14.0)	
	Malfunction		6 (18.8)	8 (15.4)	3 (2.8)	
	Self-removal		4 (12.5)	2 (3.8)	1 (0.9)	
	Change to PICC		0 (0)	0 (0)	19 (17.8)	
ICU mortality—No. of patients (%)			4 (12.5)	0 (0)	27 (25.5)	<0.001
ICU-free days—median (IQR)			2.4 (0–8.3)	11.9 (0–19.5)	6.0 (0–14.5)	0.020
Length of stay in ICU (days)—median (IQR)			24.0 (16.4–41.0)	14.5 (5.5–29.0)	15.1 (6.2–23.7)	0.003

PICC, peripherally-inserted central catheter; IQR, interquartile range; CLABSI, central line-associated bloodstream infection; ICU, intensive care unit

There were no significant differences in complication between the three groups except insertional injury (Table 3). There were two patients with late-onset CLABSI (nine and 10 days after the insertion) in the ultrasound-guided group performed by a neurointensivist. Incidence of insertional injuries was higher in CCVC insertion compared to PICC insertions (*p* = 0.038). There were eight insertional injuries in patients with CCVC (two pneumothoraxes, one hemothorax, one hemopneumothorax, four hematomas of insertional site). The ICU mortality rate was significantly higher in the patients who underwent ultrasound-guided PICC placement by a neurointensivist and CCVC placement compared to the patients who underwent PICC placement by radiologists (*p* <0.001). In addition, the ICU stay length was significantly longer in patients who underwent PICC by a neurointensivist compared to the other groups (*p* = 0.003). In addition, ICU-free days were significantly longer in the patients who underwent fluoroscopy-guided PICC placement compared to the other groups (*p* = 0.020).

## Discussion

In this study, we investigated the safety and feasibility of ultrasound-guided PICC placement by a neurointensivist compared to fluoroscopy-guided PICC placement performed by a radiologist and CCVC placement. Major findings of this study were as follows: 1) There were no significant differences in complications associated with PICC insertion between the two PICC groups. However, incidence of insertional injuries was higher in the CCVC group compared to the PICC groups. 2) The initial success rate of central line placement was significantly higher in the fluoroscopy-guided PICC placement than in the other methods. However, there was no failed central line placement after re-inserted attempt. 3) Procedure time was similar between the three groups. Duration of using central lines was shorter in CCVC group compared to PICC groups. 4) Ultrasound-guided PICC placement by the neurointensivist and CCVC placement were more frequently performed at the bedside if the patients were on mechanical ventilation and had hemodynamic instability. In addition, survival rate and ICU-free days were lower in the ultrasound-guided group and the CCVC group compared to the fluoroscopy-guided PICC group.

Neurocritically ill patients and neurosurgery patients often require central venous access using either a central venous catheter or PICC [1,5]. PICC placement does have a place in the critical care setting and it should not be expected to replace existing methods of vascular access, but may be used to provide a safe and effective alternative [12]. In the care and treatment of neurocritically ill patients, PICCs have been shown to be of particular value when used for patients undergoing parenteral nutrition, long-term antibiotic therapy, frequent blood sampling, and in patients with difficult venous access [12,21]. In addition, it is useful for infusion of drugs that requires a central line. Specifically, hypertonic saline (sodium chloride ≥3%) for management of intracranial hypertension has been administered via a central venous line because of the perceived risk of infiltration and tissue injury due to its high osmolality [22]. PICCs can also be used for central venous pressure monitoring [4,12,23]. Furthermore, PICC insertion can be carried out with no risk of pleura-pulmonary damage and with no clinically significant risk of local bleeding or hematoma, even in patients with coagulative disorders or difficult neck anatomy, and has a low risk for CRBSI compared to standard central venous catheters [1–5]. Therefore, PICCs are useful in the neuroscience ICU setting because these devices are associated with utility as central venous lines, and low-risk of insertional damage and infection [3,4]. In this study, incidence of insertional injuries was also higher in the CCVC group compared to the PICC groups.

Traditionally, PICC placement has been performed by an interventional radiologist in the interventional radiology suite under fluoroscopic guidance [2,6,7]. Intra-hospital transport of neuro-critically ill patients is required for PICC procedures performed under fluoroscopic guidance. However, intra-hospital transport of critically ill patients may be associated with risk of significant complications and should be considered an important part of the ICU risk management program [9]. It may increase the risk of complications in critically ill patients on mechanical ventilation or with hemodynamic instability [8–10]. Complications from intra-hospital transport can be life-threatening and the transport-associated mortality of critically ill patients has been reported to be 2 percent [10]. Therefore, in neurocritically ill patients, ultrasound-guided PICC placement should be performed at the bedside by a neurointensivist because of the risk of intra-hospital transport [8].

PICC placement may be performed by the specific protocol of each hospital’s PICC team [4,11]. PICCs have usually been inserted at the bedside by the vascular access team, using sterile precautions and a comprehensive prevention program [1]. For bedside insertion, ultrasound for vein localization and the modified Seldinger technique have been used, and placement was shown to be safe and feasible by use of these techniques [12,13]. In addition, PICCs were placed by a physician or experienced nurse of a specialized team in previous studies [1,4,11–13]. PICC insertion at the bedside by a vascular access team or experienced physician was safe and feasible in critically ill patients [1,4,11–13]. Therefore, ultrasound-guided PICC placement performed by an experienced neurointensivist may be safe and feasible in neurocritically ill patients [8].

PICCs are usually considered a device at low risk for CRBSI [3,4]. The incidence of CLABSI was not significantly different between the three groups. Although there were two CLABSI events in patients who underwent PICC placement by a neurointensivist, they were late-onset CLABSIs, occurring more than one week after insertion of the PICC. In addition, the ICU mortality rate and length of ICU stays were greater in patients who underwent PICC placement by a neurointensivist compared to patients who underwent PICC placement by a radiologist. Therefore, patients who underwent PICC placement by a neurointensivist might be sicker and more vulnerability to infection compared to patients who underwent PICC placement by a radiologist. In addition, there were two events of symptomatic PICC-related venous thrombosis in patients who underwent PICC placement by a neurointensivist. Although PICC placements were not performed at paretic arms in these patients, immobility according to long-term ICU stay may influence thrombotic complications.

This study had several limitations. First, it was a retrospective review of medical records. Second, PICC placement was determined by a neurointensivist or neurosurgeon rather than by a protocol-based determination. In addition, the non-randomized nature of the registry data may have resulted in selection bias. Third, there was no routine screening for central line-related venous thrombosis or pulmonary thromboembolism in this study. Finally, the neurointensivist in our hospital is a neurologist and trained to manage all aspects of critically ill patients in medical and surgical ICUs. The treatment tendency of this neurointensivist might have influenced the clinical outcomes in neurocritically ill patients. Therefore, our study may not be broadly applicable to other centers at which neurointensivists are not available because our study was conducted at a single tertiary institution with a specialized neurosurgery ICU for neurosurgical and neurocritically ill patients.

## Conclusions

Ultrasound-guided PICC placement by a neurointensivist may be safe and feasible compared to fluoroscopy-guided PICC placement by interventional radiologists and CCVC placement. Ultrasound-guided PICC placement can be performed at the bedside by a neurointensivist for neurocritically ill patients or neurosurgery patients if intra-hospital transport is contraindicated.
